# Lipocalin-2 in Stroke

**DOI:** 10.17140/NOJ-2-109

**Published:** 2015-04-17

**Authors:** Wen-Hai Chou, Guona Wang, Varun Kumar, Yi-Chinn Weng

**Affiliations:** Department of Biological Sciences, School of Biomedical Sciences, Kent State University, Kent, OH 44224, USA

**Keywords:** Lipocalin-2, NGAL, 24p3, Stroke, Reperfusion injury, Neutrophil, PKC, Phosphorylation, Biomarker

## Abstract

Stroke is a leading cause of adult disability in the United States. However, limited number of molecularly targeted therapy exists for stroke. Recent studies have shown that Li-pocalin-2 (LCN2) is an acute phase protein mediating neuroinflammation after ischemic and hemorrhagic strokes. This review is an attempt to summarize some LCN2-related research findings and discuss its role in stroke.

## INTRODUCTION

Stroke is a sudden loss of neurological function due to ischemia or hemorrhage in the brain.^1^ It is the fifth leading cause of death and a major cause of long-term disability in the United States.^2^ There are two main types of stroke: ischemic and hemorrhagic strokes. Ischemic stroke accounts for approximately 87% of all strokes and results from blockage of blood flow into the brain by thrombus or embolus. Hemorrhagic stroke, caused by rupture of cerebral blood vessels, is less common (13%) than ischemic stroke but accounts for 50% of stroke death.^3–6^ Currently, there is no proven medical or surgical treatment for hemorrhagic stroke.

Thrombolysis with fibrinolytic agents such as tissue Plasminogen Activator (tPA) is the only FDA approved therapy to reverse ischemic stroke.^7^ However, only 5% of patients receive the treatment because tPA must be given within 3 to 4.5 hours after the occurrence of stroke. Delayed treatment may increase the risk of serious side effects such as hemorrhagic transformation and reperfusion injury.^8^ Ischemia initiates cerebral infarction during ischemic stroke, but reperfusion after recanalization may promote secondary injury and worsen neurological outcomes.^8,9^ Reperfusion injury includes a series of inflammatory events with activation and infiltration of circulating neutrophils, macrophages, and T-cells into infarcted brain tissue.^10,11^ Post-stroke inflammation has detrimental effects, but may be needed for repairing processes.^11^ In order to reduce stroke-reperfusion injury and develop effective and balanced therapeutic methods, it is important to identify neurotoxic and neuroprotective molecules of post-stroke inflammation. In the acute stage of stroke (within 24 hours), infiltrating immune cells release proinflammatory cytokines (IL-1β, IL-6, TNF-α), chemokines (MCP-1, MIP-1α, IL-8), reactive oxygen species (ROS), and matrix metalloproteinase (MMP) (mainly MMP-9), which amplify neuroinflammatory responses and lead to brain edema, neuronal death, and disruption of blood-brain barrier (BBB).^8,9,11^ However, some of these molecules have a different role in the later stage of stroke (after 24 hours). For example, MMP-9 enhances ischemic brain injury, BBB leakage, and hemorrhagic transformation in the acute stage, but facilitates regeneration and remodelling of brain tissues in the later stage of stroke.^12^ Therefore, detailed mechanistic studies of post-stroke inflammation are needed.

## LIPOCALIN-2 (LCN2) IN ISCHEMIC STROKE

Lipocalin-2 (LCN2), also known as 24p3 or neutrophil gelatinase-associated lipocalin (NGAL), is a 25 kDa protein secreted from activated neutrophils.^13^ Using a chemical-genetics approach, LCN2 was identified as one of PKCδ phosphorylation substrates in neutrophils.^14–16^ PKCδ directly phosphorylates LCN2 at Thr-115, and mediates the secretion of LCN2 from activated neutrophils *in vitro* and after cerebral ischemia *in vivo*.^16^

Plasma level of LCN2 is elevated at 1–3 days in patients with ischemic stroke.^17–19^ LCN2 is acutely induced after transient middle cerebral artery occlusion (tMCAO) in rodents (a model of ischemic stroke).^20–23^ LCN2 appears in mouse sera as early as one hour, peaks at 23 hours, and diminishes by 48 to 72 hours after tMCAO.^23^ Due to the short time window for effective thrombolytic therapy, it is of great interest to diagnose stroke early and reduce the risk of cerebral hemorrhage.^24,25^ The early induction of LCN2 suggests the possibility of using LCN2 as an early blood biomarker to detect stroke.

In addition to blood plasma, LCN2 is also induced in the penumbra of ipsilateral hemispheres after tMCAO.^21–23,26^ The induction of LCN2 in mouse brain initiates at 6 hours, reaches a peak at 24 hours, and reduces at 48 hours after reperfusion. Induced LCN2 protein is identified in a subset of reactivated astrocytes, cerebral endothelial cells, and infiltrated neutrophils after tMCAO.^21,23,26^ Cerebral infarction, neurological deficits, infiltration of immune cells, BBB permeability, proinflammatory cytokines, chemokines, and adhesion molecules are reduced after tMCAO in LCN2 null mice.^21,23^ Recombinant LCN2 protein is able to stimulate neutrophil migration as well as promote cell death in primary neurons but not in astrocytes, microglia and oligodendrocytes.^21,23,27,28^ These results suggest that LCN2 is a proinflammatory mediator during the acute stage of ischemic stroke. Therefore, LCN2 inhibitors or anti-LCN2 antibodies may prove useful to reduce post-stroke inflammation and brain injury. At later time point (3 days) after ischemic stroke in rats and humans, LCN2 is expressed in injured neurons and may be released as a “help me signal” to condition microglia and astrocytes for recovery.^22^ These studies demonstrate the diverse functions of LCN2 during the acute and later stages of ischemic stroke.

## LIPOCALIN-2 (LCN2) IN HEMORRHAGIC STROKE

Hemorrhagic stroke is a devastating form of stroke with high mortality.^3–6^ There are two major types of hemorrhagic stroke: intracerebral hemorrhage (ICH) and subarachnoid hemorrhage (SAH). ICH is associated with bleeding in the brain parenchyma.^4–6^ SAH is often caused by intracranial aneurysm with blood leakage in subarachnoid space.^3^ LCN2 is induced mainly in astrocytes after rodent models of ICH and SAH.^29,30^ LCN2 induction was detected in the ipsilateral hemispheres at 1, 3, 7 days after ICH in rats and 24 hours after SAH in mice.^29,30^ Iron overload after ICH induces perihematoma edema and brain injury.^4–6^ LCN2 is capable of transporting irons through siderophore.^31–33^ Injection of iron upregulates the expression of LCN2 in the brain, while systemic treatment of an iron chelator (deferoxamine) reduces ICH-induced LCN2 upregulation.^29^ The results suggest that LCN2 may function as an important regulator of iron homeostasis after ICH. White matter injury and markers for axonal damage and myelin degradation are increased after SAH in wild type mice, but scarcely developed in LCN2 null mice.^30^ The result suggests that LCN2 may facilitate the development of white matter injury after SAH.

Several studies we summarized in this review suggest that LCN2 promotes brain injury as a proinflammatory molecule in the acute stage of stroke.^16,21,23,26,29,30^ Interestingly, LCN2 may also support the neurovascular recovery by enhancing angiogenesis and serving as a “help me signal” in the later stage of stroke.^22,34^ Therefore, a comprehensive understanding of time-dependent functions of LCN2 is a prerequisite for developing effective therapeutic interventions for the treatment of ischemic and hemorrhagic strokes.

## CONCLUSION

LCN2 has been identified as an important mediator of stroke-reperfusion injury and white matter injury after ischemic and hemorrhagic strokes. Future studies are needed to reveal the detailed mechanisms of LCN2-mediated signaling and to develop potential LCN2-based therapy.

## ABBREVIATIONS

LCN2: Lipocalin-2
tPA: tissue Plasminogen Activator
SAH: Subarachnoid hemorrhage
MMP: Matrix metalloproteinase
ROS: Reactive Oxygen Species
NGAL: Neutrophil gelatinase-associated lipocalin
tMCAO: transient Middle Cerebral Artery Occlusion
BBB: Blood-brain barrier
